# Exploring Diet-Based Treatments for Atrial Fibrillation: Patient Empowerment and Citizen Science as a Model for Quality-of-Life-Centered Solutions

**DOI:** 10.3390/nu16162672

**Published:** 2024-08-13

**Authors:** Myrthe F. Kuipers, Ronja Laurila, Maurice L. Remy, Michiel van Oudheusden, Nedra Hazlett, Sally Lipsky, Lianna L. Reisner, Debbe McCall, Natasja M. S. de Groot, Bianca J. J. M. Brundel

**Affiliations:** 1 Department of Marketing, Economics and Business Administration, Amsterdam Business School, University of Amsterdam, 1018 TV Amsterdam, The Netherlands; m.f.kuipers@uva.nl; 2 Atrial Fibrillation Innovation Platform, 1000 CE Amsterdam, The Netherlands; ronja@afiponline.org; 3 Athena Institute, Faculty of Science, VU University Amsterdam, 1081 BT Amsterdam, The Netherlands; m.l.remy2@vu.nl (M.L.R.); m.p.van.oudheusden@vu.nl (M.v.O.); 4 Allegheny Health Network, Pittsburgh, PA 15222, USA; nrhmomx3@gmail.com; 5 Plant-Based, Pittsburgh, PA 15222, USA; plantbasedpittsburgh@gmail.com; 6 Plant Powered Metro New York, New York, NY 10025, USA; lianna.levine.reisner@ppmny.org; 7 Journal of Atrial Fibrillation and Electrophysiology, Overland Park, KS 66209, USA; debbe.mccall@gmail.com; 8 Department of Cardiology, Erasmus Medical Center, 3015 GD Rotterdam, The Netherlands; n.m.s.degroot@erasmusmc.nl; 9 Department of Physiology, Amsterdam UMC, Location Vrije Universiteit, Amsterdam Cardiovascular Sciences, Heart Failure and Arrhythmias, 1081 HZ Amsterdam, The Netherlands

**Keywords:** atrial fibrillation, whole foods, plant-based diet, risk factors, obesity, patient empowerment, Mediterranean diet, quality of life, citizen science, cocreation, patient-reported outcome measures

## Abstract

Atrial fibrillation (AF) is the most common heart rhythm disorder in the Western world. Between the years 2010 and 2019, the global prevalence of AF rose from 33.5 million to 59 million, highlighting the importance of developing equitable treatments for patients. The disease is associated with symptoms such as palpitations, dizziness, fatigue, shortness of breath, and cognitive dysfunction. In addition, AF increases the risk of developing a stroke and heart failure. Despite new insights into risk factors that can lead to the development of AF, the success of current treatments is suboptimal. Numerous risk factors, such as hypertension, diabetes, and obesity, have been associated with the development and progression of AF. As these can be lifestyle-related risk factors, lifestyle modification may be a solution to reduce AF-related symptoms as well as episodes. Research results show that certain dietary changes can reduce AF and numerous risk factors for AF. Increasing attention is being given to Mediterranean and whole, plant-based eating patterns, which emphasize eating grains, legumes, vegetables, fruits, and nuts, while excluding most—or all—animal products. Hence, what are the beneficial aspects of a Mediterranean and plant-based diet which consists mainly of unprocessed foods? In the current review, we discuss the outcomes of diet-based treatments. Moreover, other diet-related treatments, brought up by patient initiatives, are highlighted. These patient-initiated studies include L-glutamine and electrolytes as options to manage AF. Also, we highlight the emerging importance of valuing patient needs and a quality-of-life-centered approach to medicine. As indicated by recent studies and patient experiences, citizen science can create inclusive solutions that lead to patient empowerment and a holistic approach for AF management.

## 1. Introduction

Atrial fibrillation (AF) is the most common cardiac arrhythmia of the 21st century. AF is most prominent in the Western world and affects approximately 60 million people worldwide [1,2]. People with AF are five times more likely to suffer from a stroke and associated life-changing damage compared to healthy people. In addition, AF increases the risk of developing heart failure [1]. Numerous risk factors, such as hyperthyroidism, diabetes, obesity, and hypertension, have been associated with AF (Table 1). Also, specific triggers, which have been commonly reported by patients and are very individual, may drive AF episodes [1]. These risk factors and triggers are often related to the lifestyle in Western countries, as illustrated by a recent study from the MOnica Risk, Genetics, Archiving and Monograph (MORGAM) consortium that included a large cohort of adult and elderly European men and women [3]. The research, involving 66,951 participants, revealed that the risk of atrial fibrillation (AF) was predominantly linked to body mass index, hypertension, and a history of stroke or myocardial infarction, accounting for 30–40% of the overall AF risk. Interestingly, body mass index significantly contributed to the burden of AF in individuals aged 40–69 years, while hypertension was more prevalent in those aged 60 years and older. Additionally, the risk of AF associated with a history of myocardial infarction or stroke increased marginally with age. This raises the question whether an adjustment in this lifestyle helps to reduce the risk factors for AF and, therefore, possibly the symptoms associated with AF.

While various lifestyle interventions advocate for combining lifestyle changes with risk factor management in clinical practice, there is still a lack of randomized clinical trials that explore the long-term effectiveness and consistency of these benefits [11].

So far, beneficial lifestyle interventions, including weight loss, moderate exercise, mind–body exercise, and obstructive sleep apnea therapy, have been investigated [12]. In addition, studies have revealed that specific diets—notably, the Mediterranean and plant-based diets—may help to reduce the AF burden. In the current review, we discuss the outcomes of diet-based treatments. Moreover, other lifestyle treatments are highlighted, as some are brought up by patient initiatives. These patient-initiated studies include L-glutamine [13], magnesium taurate, and electrolytes as options to manage (paroxysmal) AF [14]. Also, the emerging importance of valuing patient needs and a quality-of-life-centered approach to medicine is being highlighted. As indicated by recent studies and patient experiences, citizen science can create inclusive solutions that lead to patient empowerment and a holistic approach for AF management.

## 2. Reducing AF Risk Factors through Diet

### 2.1. The Mediterranean Diet

Addressing the reversible risk factors for AF could prove effective in both primary and secondary AF prevention. Various diets have demonstrated potential to decrease the impact of AF. For example, the Mediterranean diet primarily consists of a plant-based diet, with contributions from animal-based products largely consisting of fish and poultry, along with a limited quantity of dairy products. This dietary approach focuses on incorporating healthy fats, whole grains, a variety of fruits and vegetables, as well as beans, nuts, and seeds. While limiting ultra-processed foods and meat, this eating plan encourages the consumption of fresh, unprocessed foods, a variety of fruits and vegetables, and whole foods. The PREDIMED study revealed that incorporating extra-virgin olive oil into a Mediterranean diet significantly lowered the occurrence of AF [15]. The ongoing PREDIMAR trial is evaluating a comparable intervention for secondary prevention. A recently published PREDIMED study demonstrated that Mediterranean diet adherence results in lower epicardial adipose tissue, with high levels of epicardial adipose tissue (>135 g) associated with persistent AF [16]. The findings indicate a potential health benefit of a Mediterranean diet in reducing the incidence of AF as well as potential risk factors associated with AF. However, the results of the PREDIMED trial have been debated due to possible protocol deviations; consequently, repeated trials are needed for more conclusive data [17]. Nonetheless, studies seem to indicate that a Mediterranean food pattern can aid in diminishing the negative effects of AF as well as reducing major cardiovascular events, inflammation, and diabetes. In addition, a preventative effect of the diet on atherosclerosis and coronary heart disease has been proposed [18,19].

### 2.2. Whole Foods Plant-Based Diet

Even though the Mediterranean diet incorporates some animal foods, a whole foods plant-based diet is more restrictive in its avoidance of animal products and processed foods. Currently, a whole foods plant-based diet is a treatment option being explored at length due to its positive outcomes for patients with cardiovascular disease that can be managed with lifestyle alterations (Box 1). The diet places a strong emphasis on whole, unprocessed plant foods such as vegetables, fruits, beans, whole grains, legumes, and herbs and spices (Table 2). Research on plant-based diet patterns and their relationship to the risk of developing AF is limited to some extent, but, recently, researchers have paid more attention to reducing risk factors through lifestyle modifications. In this context, dietary adjustment is of utmost importance.

Box 1 **Patient experience: how a plant-based diet can change living with atrial fibrillation.**

“When I was diagnosed with atrial fibrillation years ago, I had no idea that a lifestyle change, and especially a change to a plant-based diet, could have such a dramatic impact on my atrial fibrillation. If someone had asked me at the time, do you eat healthy? Then I would have answered ‘yes, absolutely’. I ate a varied diet every day including vegetables, fruit, dairy products, and meat. Meanwhile, my cholesterol levels were high. This runs in my family and there was nothing I could do about it myself. In addition, I had prediabetes and high blood pressure since my first pregnancy. Again, no one mentioned my diet in any context other than avoiding sodium, which I did.

In 2014, after having atrial fibrillation for 12 years, and prior to having a radiofrequency ablation, I received an email from my treating cardiologist and electrophysiologist. He offered a hands-on course for 50 of his patients. He said the Complete Health Improvement Program (CHIP) course, 16 weeks in total, would improve my health. During the weekly classes, I learned how to avoid meat and dairy in my diet. I also learned how to put together a complete diet with fruits, vegetables, and whole grains. These adjustments have radically changed my life. My energy levels immediately increased, my sleep improved, and most importantly, my cardiac health improved! Monthly laboratory results from blood samples showed a clear pattern: my cholesterol and glucose levels recovered and have shown a normal pattern since I started following a plant-based diet. One year later, after significantly improving my overall health, I had a catheter ablation in 2015. Over the past 9 years I have been completely atrial fibrillation free. Healthy lifestyle, in terms of diet, stress management, exercise and restorative sleep, is the cornerstone of long-term ablation success. A plant-based lifestyle has been the keystone of this regimen that has led to my Afib free life.” Nedra

An illustrative case of how a plant-based diet can lower risk is demonstrated in the research conducted by Pathak et al. [21]. Their study found that AF patients who lost more than 10% of their body weight experienced a sixfold increase likelihood of remaining AF-free, compared to patients who lost less than 10% during the same timeframe. Sustained weight loss also ensures that patients remain in a normal sinus rhythm for a longer period of time. In addition, recent studies have shown that a diet consisting of a lot of ultra-processed food (i.e., food products made in a factory with additives such as salt, sugar, fats, modified starch, and E numbers) is connected with the development of AF. However, the opposite effect has been found with a plant-based diet, as this diet is not associated with the development of AF [22,23]. 

Due to the reported beneficial effect of a plant-based diet on cardiac health, this diet has gained increasing attention from the medical community. Studies show that plant-based diets, which have been considered as three meals per day, help decrease many common risk factors for AF, including hypertension, hyperthyroidism, diabetes, and obesity (Table 3) [24,25]. Plant-based diets may also help lower emerging and understudied risk factors, like inflammation. In addition, a complete plant-based diet contains high magnesium levels [26,27,28]. Given that reduced serum magnesium levels are somewhat linked to the onset of AF in those without preexisting cardiovascular issues, adopting a plant-based diet could offer a beneficial approach for these individuals.

## 3. Modifiable Risk Factors and the Effect of a Plant-Based Diet

### 3.1. Hypertension

Notably, adopting plant-based diets can result in a significant decrease in average blood pressure [31]. A meta-analysis showed that consumption of vegetarian diets was associated with lower mean systolic blood pressure (−6.9 mm Hg; 95% CI, −9.1 to −4.7; *p* < 0.001) and diastolic blood pressure (−4.7 mm Hg; 95% CI, −6.3 to −3.1; *p* < 0.001), compared to the consumption of omnivorous diets [32]. Plant-based diets also play an important role in the prevention of hypertension [31]. A cross-sectional study involving 11,004 British men and women, which compared meat eaters, fish eaters, vegetarians, and vegans, revealed that the vegan group had the lowest incidence of hypertension [34]. 

Mechanisms through which a plant-based diet may lower blood pressure include enhanced vasodilation (relaxation of blood vessels), anti-inflammatory properties, higher antioxidant levels, increased potassium intake, and decreased blood viscosity. Considering these results and the connection between hypertension and AF, advising patients with either condition to adopt a plant-based diet appears to be a prudent recommendation.

### 3.2. Diabetes and Obesity

Obesity and diabetes are also significant risk factors that independently contribute to the likelihood of developing AF [12]. Findings from the ARIC study showed that obesity and overweight (characterized by a BMI ≥ 25 kg/m²) were responsible for roughly 18% of new cases of AF [57], while diabetes accounted for about 3% of these new AF cases [57]. In this scenario, plant-based nutrition is particularly significant. Results from the Adventist Health Study-2 revealed that the incidence of type 2 diabetes among those adhering to a vegetarian diet was roughly 50% lower compared to those on an omnivorous diet [24,38]. Besides vegetarian diets being associated with reduced diabetes, these diets are also linked to lower body mass index. This positive effect of vegetarian diets on diabetes and healthy weight has been confirmed by several additional studies [33,54]. Interestingly, in short- and long-term studies, weight loss in overweight and obese people has been associated with improvement in AF burden and AF severity [21,58]. Research even showed a relationship between the degree of weight loss and the effect on AF-free episodes. For example, patients with less than 3% weight loss experienced 40% fewer AF attacks; patients with between 3% and 10% weight loss experienced 66% fewer AF attacks; and patients with more than 10% weight loss experienced as much as 86% fewer AF attacks [21,59]. A plant-based diet can be a useful instrument to help individuals attain and maintain a healthy normal weight.

### 3.3. Inflammation

There is increasing evidence for a close link between AF and inflammation [47]. Inflammation can contribute to both the progression and upholding of AF. Components of the inflammatory response can change the electrical currents in the atria, causing AF [47]. A plant-based diet is known to be rich in anti-inflammatory and antioxidant components. Several studies have shown that diets rich in unrefined plant foods reduce the inflammatory factor CRP [35,60]. A study found that omnivores had notably higher CRP levels compared to vegans, with averages of (1.1 mg/L versus 0.5 mg/L, respectively) [61]. Moreover, a recent meta-analysis showing the connection between a vegetarian diet and inflammatory biomarkers, concluded that a vegetarian diet may be a beneficial long-term method to managing inflammation [49].

### 3.4. Heart Failure and Coronary Artery Disease

Additional key risk factors for AF encompass heart failure and coronary artery disease (CAD). Plant-based diets have been demonstrated to effectively address risk factors and can even stop or reverse CAD [52,62,63,64]. The Lifestyle Heart Trial revealed that over 80% of patients with heart disease who adhered to a plant-based diet program exhibited varying degrees of atherosclerosis improvement, and 91% reported a decrease in angina episodes [53]. A second larger study involving almost 200 patients found the same favorable results in CAD patients. Approximately 198 participants received guidance on plant-based nutrition and were instructed to completely remove, fish, meat, and added oils, from their daily diet. Over the course of the four-year follow-up, 99.4% of those adhering to a whole-food plant-based diet did not experience any major cardiac events. Angina pectoris disappeared or improved in almost 93% of participants. The possible underlying mechanisms include preventing damage to vascular endothelial cells and preventing LDL oxidation, along with oxidative inflammation through improved antioxidant intake [52,54].

### 3.5. Epicardial Adipose Tissue

Epicardial adipose tissue (EAT) is associated to the development and severity of AF [59]. The thickness of EAT has been connected with the incidence and severity of AF. Given its anatomical contiguity with the adjacent left atrium, EAT has been implicated in the pathogenesis of AF [65]. Targeting EAT through interventions such as exercise, diet, bariatric surgery, or pharmaceutical approaches has been the subject of a systematic review and meta-analysis, indicating the potential therapeutic significance of addressing EAT in the context of AF [56].

## 4. Patient Empowerment as a Model for Quality-of-Life-Centered Approaches

Based on the shown evidence, physicians may more often recommend a Mediterranean or plant-based diet to their AF patients. Moreover, certain supplements may also play a beneficial role in reducing the burden of AF, especially if, due to, e.g., dietary restrictions, an exhaustive implementation of a plant-based diet is not feasible. Improving one’s health-related quality of life through a simple lifestyle adjustment enables patients to be in more control of their treatments and leads to a patient-tailored and -centered therapy.

In the design of patient-tailored treatment, patients are key participants in finding easy-to-implement solutions. Patient experiences have been proven to be a valuable tool for gaining insight into individual patient values and how the health-related quality of life of different demographics can be maximized [66], especially given the unequal effectiveness of treatments among patients and the beneficial effects of creating tailored treatment options to address specific needs [1].

### 4.1. L-Glutamine and AF

Research has shown that AF contributes to the exhaustion of the atria due to the rapid activation rate of the atrial cardiomyocytes. Various studies have shown that agents that increase the cardioprotective heat shock proteins (HSP) in the atria may help patients prevent, reduce, and even recover from AF [67,68,69]. An observant AF patient read in an article that the dietary supplement L-glutamine, available in any drugstore, can also increase HSP in heart muscle tissue. Based on this observation, the AFIP foundation designed a study founded on the idea of co-creation between patients and researchers. The outcomes of this study showed the beneficial effect of L-glutamine on blood-based HSP levels and metabolite levels related to energy, nucleotides, and amino acid production [13]. Without the patient-led initiative, the positive effects of L-glutamine might have never been properly explored. 

### 4.2. Electrolytes and AF

Another instance of patient initiative towards finding a custom treatment plan was in the instance of electrolytes and AF management. An AF patient came across several studies investigating the effect of an electrolyte deficiency on the development of AF (Box 2). In particular, the patient noted that low potassium, magnesium, calcium, and Q10 can result in higher AF risk [70]. Also, exercising and ingesting beverages that deplete magnesium stores (alcohol and coffee) seemed to correlate with AF episodes. This led the patient to experiment with how electrolytes affect AF episodes. After a self-trial period of finding best practices, the patient managed to create a tailored treatment that resulted in the suppression of all AF episodes for at least 9 months. Other than occasional palpitations, the patient noticed almost no symptoms and reported an increase in health-related quality of life offered by this unconventional treatment. The potential of magnesium supplementation to prevent and/or treat AF has been acknowledged by the AF patient community. Many individuals with AF have shared on patient platforms that magnesium glycinate and magnesium taurate have been beneficial in managing their condition [1]. The primary physiological roles of magnesium include enzyme activity, protein transport, and serving as an essential component of all ATP-utilizing systems. Therefore, low dietary intake of magnesium has been linked to a 50% higher risk of new-onset AF [71]. A meta-analysis of 20 randomized controlled trials has suggested that prophylactic magnesium supplementation may help prevent post-operative AF onset, whereas a systematic review and meta-analyses on incident on new onset AF indicate no effect. However, the studies included represented a large variety in study population that may have hindered the outcomes [72]. Therefore, further studies on optimal dose, timing, and type of magnesium are required to obtain conclusive answers.

Box 2 **Patient experience: electrolytes help stop atrial fibrillation.**

“Last year in September (2022) I had my first episode of atrial fibrillation. From then on, I experienced new episodes every 2 to 3 weeks. My GP referred me to a cardiologist, who indicated that I had paroxysmal atrial fibrillation. The cardiologist prescribed a pill that I could take during an attack. But usually, an atrial fibrillation attack did not last long for me: about 4 to 5 h and it was not necessary to take the pill.

I have always been interested in lifestyle and nutrition, and I therefore started looking for possible causes of atrial fibrillation, as well as solutions to stop this rhythm disorder. I came across several studies on the internet that showed that a lack of electrolytes in the body can play a role in the development of atrial fibrillation. In particular, low values in potassium, magnesium, calcium, and Q10 were mentioned in these studies. In addition, coffee, alcohol, and strength training can deplete the magnesium stores in your body. This research compelled me to experiment with electrolyte values.

After a period of trying different things, I finally found what works for me. Since January this year (2023) I have been taking 200 mg of Q10 and 300 to 400 mg of magnesium lactate daily as well as a pack of fresh coconut water weekly. This seems to help me enormously!

Last January, I experienced 2 very mild attacks of atrial fibrillation, and since then (9 months) no more attacks. I also no longer notice my heart skipping a beat. In the past, I occasionally had some irregularities in my heartbeat between attacks of atrial fibrillation. For me, adding electrolytes to my diet works particularly well. I hope this will apply to more people with atrial fibrillation.” Arrien

## 5. Patient-Tailored AF Solutions through Citizen Science

Citizen science is a concept that is rising in popularity, especially in healthcare, due to the translational nature of citizen-science-based research [73,74]. Citizen science bridges the divide between patients, researchers, and health practitioners, allowing patients to become major stakeholders in research and development (R&D) processes [75]. Within healthcare R&D and guideline development, particularly for specific patient populations (e.g., sex, age, socio-economic status), citizen science enables more personalization for and equity among patients [76]. Furthermore, it can empower patients in dealing with their condition.

An example of citizen science is the implementation and improvement of personalized treatment plans via the AFIP foundation. This online health community consists of AF patients, family members, caregivers, and researchers. By sharing lived experiences, the community not only experiences peer learning, but also new research projects are collectively initiated by both researchers and AF patients. In 2020, the AFIP foundation conducted a poll on individual triggers for AF (N = 1051). Stress was reported as the most frequent trigger for AF episodes (23.8%), with food habits coming in second place (21.5%). Among food habits, the most common triggers were a “full stomach” (N = 75), alcohol (N = 40), and coffee (N = 10).

**Figure 1:**
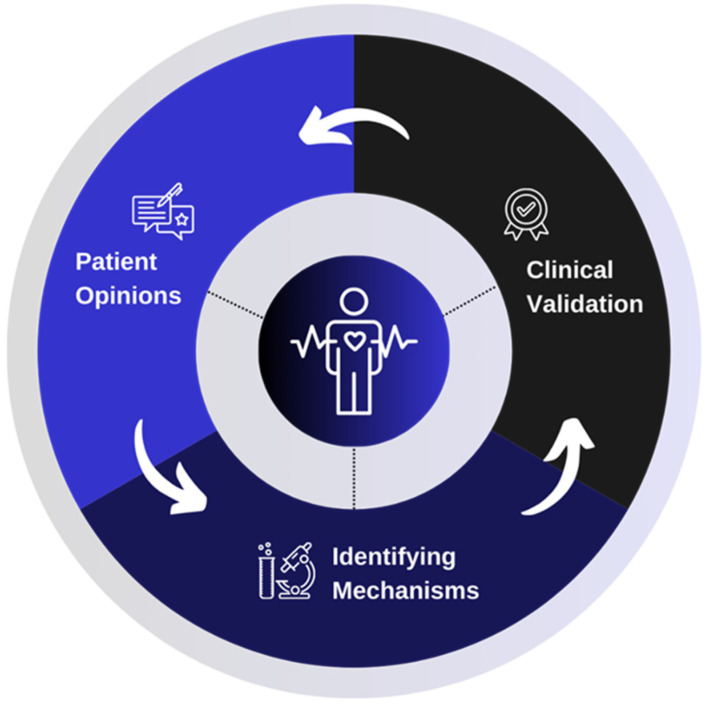
CIRCULAR patient and researcher collaboration model. Patients’ opinions are collected and form the base of research on mechanisms and clinical validation. After finalization of these phases, outcomes are given back to the patients. Hence, patients remain at the center of every stage of the model.

Based on these patients’ reported outcomes, a new international AF network named CIRCULAR was created in 2022 as part of the Dutch Research Agenda. This network consists of universities, colleges, companies, non-profit organizations, and, most importantly, patients. As part of this project, patients are being approached via social media to become part of the community and share personal insights with the AFIP foundation(e.g., their most prominent triggers for an AF episode). Based on patients’ contributions, professionals prioritize the most reported triggers, followed by a pre-clinical investigation of the molecular mechanism underlying trigger-induced AF in animal-free model systems. Based on the outcomes of the lab studies, new treatment options will be developed and validated using the latest techniques, such as AF-on-a-chip approaches. Throughout the CIRCULAR project, patients remain at the center in every stage of the cycle. Figure 1 depicts how patients are integrated within this holistic AF research.

In addition to academics taking a citizen science approach, such as in CIRCULAR, citizens themselves are actively initiating changes for health improvement. An interesting example is the Plant-Powered Metro New York movement that, in early 2019, was established by volunteer leaders from five PlantPure Communities networks in the Bronx, Manhattan, Queens, and Long Island, who united to enhance coordination, support, and impact in the metro area, aiming for healthier communities through whole foods and plant-based nutrition. Amongst others, through a program with weekly educational and mentorship sessions, the self-reported quality-of-life and (mental) health outcomes improved [77]. Another example is Plant-Based Pittsburgh, which was founded by Sally Lipsky, who educated herself on the power of food in healing and protecting from disease while she was undergoing treatment for late-stage cancer. Her organization provides education and support for individuals seeking to adopt and sustain a healthy plant-based lifestyle. Nedra Hazlett, a Plant-Based Pittsburgh board member, became a totally plant-based eater in 2014. Nedra had an ablation in 2015 and has been AF free since then. Noteworthy is Nedra’s ongoing devotion to a healthy lifestyle centered on the adoption of whole foods plant-based eating (Box 1).

While an early diagnosis of AF is important to improve the success rates of medical treatment, health professionals may include programs to optimize lifestyle based on patient-reported triggers. In line with that, the American Heart Association prepared a scientific statement on lifestyle and risk factor modification to reduce AF. The statement highlights implementation of strategies, care pathways, and education to make the first steps towards an AF-free society [12].

## 6. Conclusions

Atrial fibrillation is the most common heart rhythm disorder in Western countries. Addressing risk factors and other underlying conditions that predispose individuals to AF is an emerging concept, which warrants better implementation into routine clinical care. The management of risk factors is an essential pillar of AF care, in addition to heart rhythm and rate control, and appropriate anticoagulation. An increasing number of studies show that plant-based diets can help prevent and reduce many of the risk factors of AF.

Plant-based diets emphasize whole grains, legumes, vegetables, fruits, nuts, and seeds while excluding most (or all) animal products. Diets based on whole-plant foods not only maximize protective foods but also exclude potentially harmful animal foods that are high in saturated fat [25]. In short, plant-based diets reduce lifestyle-caused diseases, including hypertension, diabetes, and obesity, as well as cardiovascular disease. Plant-based diets, therefore, appear to be an important factor for preventing and reducing risk factors of AF and, hence, possibly AF episodes themselves.

However, for those patients for whom a plant-based diet is not an option due to, for example, allergies or other dietary restrictions and lifestyle, diet-based solutions should still be explored. Hereby, patient initiatives and the use of citizen science can aid in discovering and creating equitable and personalized healthcare solutions. Examples of more unconventional treatment options brought up by patients may help to manage AF symptoms. Furthermore, quality-of-life-centered approaches and customizable care, as part of citizen science, could promote diversity and inclusivity in healthcare.

## Figures and Tables

**Table 1:** Overview of the main and emerging risk factors and patient-reported common triggers for AF.

***Main risk factors [4,5]:*** Male gender Age Hypertension Coronary artery disease Heart failure (both with reduced and preserved ejection fraction) Heart valve disease Diabetes Overweight or obesity Hypothyroidism and hyperthyroidism Cancer treatment [6] ***Emerging and less studied risk factors:*** Inflammation [7] Obstructive sleep apnea [5] Chronic kidney disease [8] Sedentary lifestyle [4,5] Endurance sport [4,5] ***Selection of patient-reported triggers for AF (via AFIP Foundation):*** Alcohol [7] Caffeinated (soft) drinks (coffee, black tea, energy drinks) [7] Monosodium glutamate (MSG, E621) Low electrolyte levels (including magnesium, potassium, calcium) in blood [9] Low vitamin D levels in blood [10]

**Table 2:** Key differences between the whole foods plant-based diet and the Mediterranean diet.

Type of Diet	Included	Excluded
Whole Foods Plant-Based Diet	Whole grains, fruits, vegetables (starchy and non-starchy), legumes (peas, beans and lentils), nuts and seeds (from Center for Nutrition studies).	The diet does not include dairy products, meat products, fish, and eggs. Also, minimal added oils, fats, sugar, and processed foods.
Mediterranean Diet	Poultry, fish, grains, seeds, and healthy oils like extra virgin olive oil. Also, low-fat dairy products. The diet recommends limiting poultry and dairy to 4 servings weekly [20].	The diet recommends avoiding red meat, processed sugary foods, and high-fat foods.

**Table 3:** Effect of plant-based diet on modifiable risk factors for AF (adapted from [29]).

Modifiable Risk Factor	Result of a Plant-Based Diet	Physiological Effects
Hypertension [12,30]	Reduced hypertension and risk of hypertension [31,32,33,34].	Improved vasodilation [21,31]; increased potassium intake [35,36]; reduced blood viscosity [31,37].
Diabetes [12,30]	Improved glycemic control and reduced insulin resistance [38,39,40].	Reduced glycation end products and low saturated fat content; reduced lipotoxicity [41,42,43].
Obesity and obstructive sleep apnea [12,30]	Reduced risk of obesity; suitable diet for weight loss [44,45,46].	High fiber and low-fat content of plant foods. Reduced energy density and increased energy consumption [44].
Systemic inflammation [12,47,48]	Decreased factors of the inflammatory response (hsCRP) [49].	Increased in anti-inflammatory and antioxidant components [50]. Absence of pro-inflammatory fats [51].
Coronary artery disease [12,30]	Prevention and gradual recovery from atherosclerosis and coronary heart disease [52,53].	Prevention of vascular endothelial cell damage [52,54]. Prevention of LDL oxidation and oxidative inflammation through increased antioxidants [54].
Epicardial adipose tissue (EAT)	Weigh loss reduced EAT [55,56].	Reducing EAT can help maintain sinus rhythm and reduce AF burden [55].
